# Diagnostic value of echocardiography in paracardiac cystic lesions: 43 cases from one single medical center

**DOI:** 10.1007/s10554-021-02180-9

**Published:** 2021-02-23

**Authors:** Jinfeng Liu, Qing LV, Jing Wang, Li Zhang, Mingxing Xie, Yali Yang

**Affiliations:** 1 Department of Ultrasound, Union Hospital, Tongji Medical College, Huazhong University of Science and Technology, Wuhan, 430022 China; 2 Department 3 of Echocardiography, Beijing Anzhen Hospital, Capital Medical University, Beijing, 100029 China; 3 Hubei Province Key Laboratory of Molecular Imaging, Wuhan, 430022 China

**Keywords:** Echocardiography, Paracardial cystic lesions, Pericardial cyst, Diagnosis

## Abstract

Paracardial cystic lesions (PCLs) are rare, benign lesions and may occur in any part surrounding the heart. It covers a variety of pathological types, including pericardial cysts, thymic cysts, bronchogenic cysts and so on. The aim of this study was to summarize the diagnostic value of echocardiography in different pathological types of the PCLs. Echocardiographic features of 43 consecutive PCL patients treated at the Union Hospital from January 2002 to December 2017 were compared and analyzed with their surgical and pathological findings retrospectively. The PCLs included 19 pericardial cysts, 12 thymic cysts, 7 bronchogenic cysts, 3 cystic teratomas, 1 enteric cyst and 1 lymphangioma. Among them, 29 cases (67.4%) were accurately diagnosed by echocardiography and 14 cases (32.6%) were missed the diagnosis. All diagnosed cysts were showed as thin-walled, monolocular, echo-free structures without blood flow signals in echocardiographic images. 4 patients had compression of the heart and great vessels caused by cysts. In addition, 4 intracardiac lesions were diagnosed by echocardiography and the results were further confirmed in surgery. Echocardiography is of great value in the diagnosis of paracardiac cystic lesions as well as combined intracardiac lesions. Differential diagnosis could be mainly made based on the location of the lesions.

## Introduction

Paracardial cystic lesions (PCLs) are defined as the cystic structures adjacent to the heart [1]. The PCLs are rare, benign lesions and may occur in any part surrounding the heart [2, 3]. They are generally asymptomatic and usually discovered incidentally in imaging examination [2]. At present, echocardiography is a widely used imaging technique in clinical practice and it is inevitable to encounter the PCLs in the examination [4]. Previous reports with regard to the PCLs by echocardiography are mainly case reports and there are few studies covering the entire range of these lesions. This is a retrospective study to demonstrate the diagnostic value of echocardiography in different types of the PCLs based on our experiences along with a literature review.

## Materials and methods

### Patient population

Echocardiographic results of the patients definitely diagnosed as PCLs were retrospectively investigated between January 2002 and December 2017 at Union Hospital, Wuhan. The diagnosis of PCLs was based on imaging modalities [including echocardiography, computed tomography (CT) or magnetic resonance (MR)] and further validated by pathological results after surgical resection. It encompassed a wide spectrum of pathology, including pericardial cyst, thymic cyst, bronchogenic cyst, enteric cyst, cystic teratoma and lymphangioma. The exclusion criteria of the study were patients suspected with PCL by echocardiographic examination but not confirmed by surgical resection. In addition, cystic thymomas were excluded from this study since these diseases may be associated with neoplastic lesions.

All patients were analyzed for sex, age, symptoms, location, size, pathological types of cysts and occurrence of cardiovascular compression. The echocardiographic performances were compared with the surgical results and characteristics of other imaging modalities (CT or MR).

### Echocardiography

All the patients underwent a complete transthoracic echocardiographic examination using a commercial ultrasound system (GE Vivid 7, Vingmed, Horten, Norway; Philips IE 33 and Philips IE Elite, Andover, MA, USA; Acuson Sequoia C256, Mountain View, CA, USA) with a 3–7.5 MHz transducer. The key planes included parasternal long-axis and short-axis views of the left ventricle, short-axis view at the level of great arteries, apical four-chamber and five-chamber views and some modified, nonstandard views showing the PCLs. In addition to echocardiography, most patients also underwent CT and/or MR examination. The CT examination was mainly non-contrast CT, and sometimes was contrast-enhanced CT. The MR scan included routine sagittal, coronal and transverse T_1_-weighted imaging and T_2_-weighted imaging, and sometimes contrast enhanced examination.

Because the heart was the main acoustic window in echocardiography, in this study, we described the location of the PCLs based on their relative position to the heart instead of their location in the mediastinum. The locations of the cysts were divided into five main areas relative to the heart: the anterior region, the posterior region, the left region, the right region and the upper area adjacent to the great arteries.

### Statistical analysis

All quantitative results were presented as mean ± SD. The missed diagnosis rates in different locations of echocardiography were compared by Fisher exact probability method of R × C tables. Differences were considered to be significant if the *P* value was < 0.05 (2 sided).

## Results

A total of 43 cases were enrolled and analyzed including 21 males (48.8%) and 22 females (51.2%). The mean age was 42.0 ± 17.3 years (2 months to 70 years). 29 patients (67.4%) were symptomatic. The common symptoms were cough (25.6%), chest pain (25.6%) and chest distress (23.2%). The other 14 patients (32.6%) were asymptomatic and the PCLs were discovered accidentally in physical examination.

The pathologic distribution of the PCLs was as follows: 19 pericardial cysts (44.2%), 12 thymic cysts (27.9%), 7 bronchogenic cysts (16.3%), 3 cystic teratomas (7.0%), 1 enteric cyst (2.3%) and 1 lymphangioma (2.3%). The cyst sizes were variable and the maximal diameter was ranging from 2 to 10 cm. Details were shown in Table 1.

**Table 1:** Clinical data and diagnostic results of 43 patients with paracardiac cystic lesions

	Pericardial cyst	Thymic cyst	Bronchogenic cyst	Cystic teratoma	Enteric cyst	Lymphangioma
Cases (n/percentage)	19 (44.2%)	12 (27.9%)	7 (16.3%)	3 (7.0%)	1 (2.3%)	1 (2.3%)
Gender (M/F)	11/8	6/6	3/4	0/3	1/0	0/1
Age (years)	41.0 ± 10.7	49.2 ± 16.6	41.4 ± 26.4	35.0 ± 20.1	0.5	43.0
Symptoms	Cough (5) Chest distress (4) Chest pain (4)	Chest distress (5) Chest pain (3) cough (2)	Chest pain (3) Chest distress (1) Cough (1)	Cough (1) Chest pain (1)	Cough (1)	Cough (1)
Positions^a^ (n/percentage)	Right 13 (68.4%) left 3 (15.8%) Upper 2 (10.5%) Anterior 1 (5.3%)	Upper 8 (66.7%) Right 3 (25%) Left 1 (8.3%)	Upper 4 (57.1%) anterior 2 (28.6%) posterior 1 (14.3%)	Right 2 (66.7%) Left 1 (33.3%)	Posterior 1 (100%)	Right 1 (100%)
Maximal diameter (cm)	5.9 ± 2.0	7.4 ± 2.4	4.4 ± 1.8	7.7 ± 1.5	8	3
Detected by echo (n/perentage)	14 (73.7%)	8 (66.7%)	4 (57.1%)	2 (66.7%)	1(100%)	0 (0)
Cardiovascular Compression	None	None	RPA (1) LA and PA (1)	RA (1)	LA and PV (1)	None
Intracardiac findings (n)	ASD (1) MVP (1)	PFO (1)	PFO (1)	None	None	None

*ASD* atrial septal defect, *LA* left atrium, *MVP* mitral valve prolapse, *PA* pulmonary artery, *PFO* patent foramen ovale, *PV* pulmonary vein, *RA* right atrium, *RPA* right pulmonary artery

^a^The relative position to the heart

**Fig. 1:**
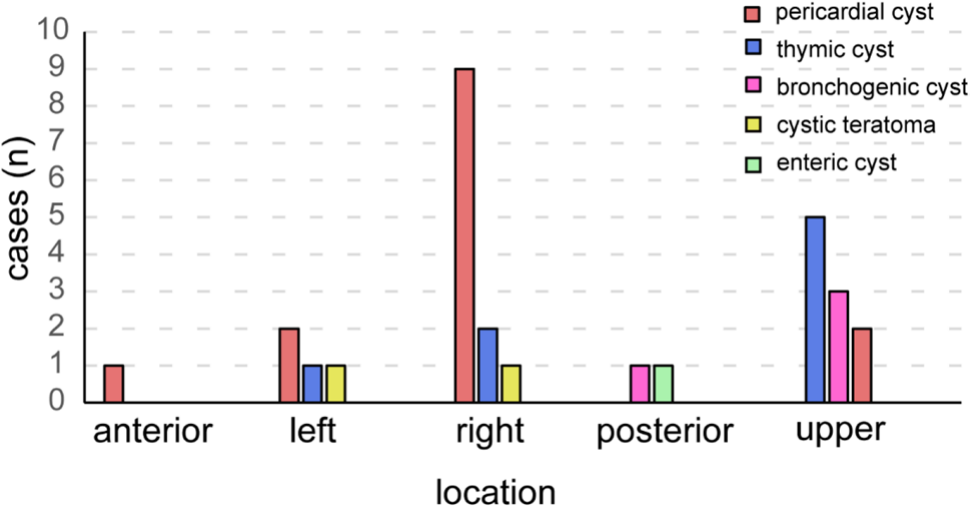
Pathological distribution in each region of 29 PCLs patients diagnosed by echocardiography

Of all the patients, 37 cases underwent CT examination and 10 underwent MR examination. Either CT or MR clearly demonstrated the cyst lesions in all cases with a 100% positive rate. In contrast, only 29 cases were detected and 14 cases were missed the diagnosis by echocardiography with a 67.4% positive rate for the PCLs. The missed diagnosis rates of echocardiography in the five regions were not significantly different (*P* < 0.05). The pathological distribution of the 29 diagnosed PCLs in each region to the heart was shown in Fig. 1.

**Fig. 2:**
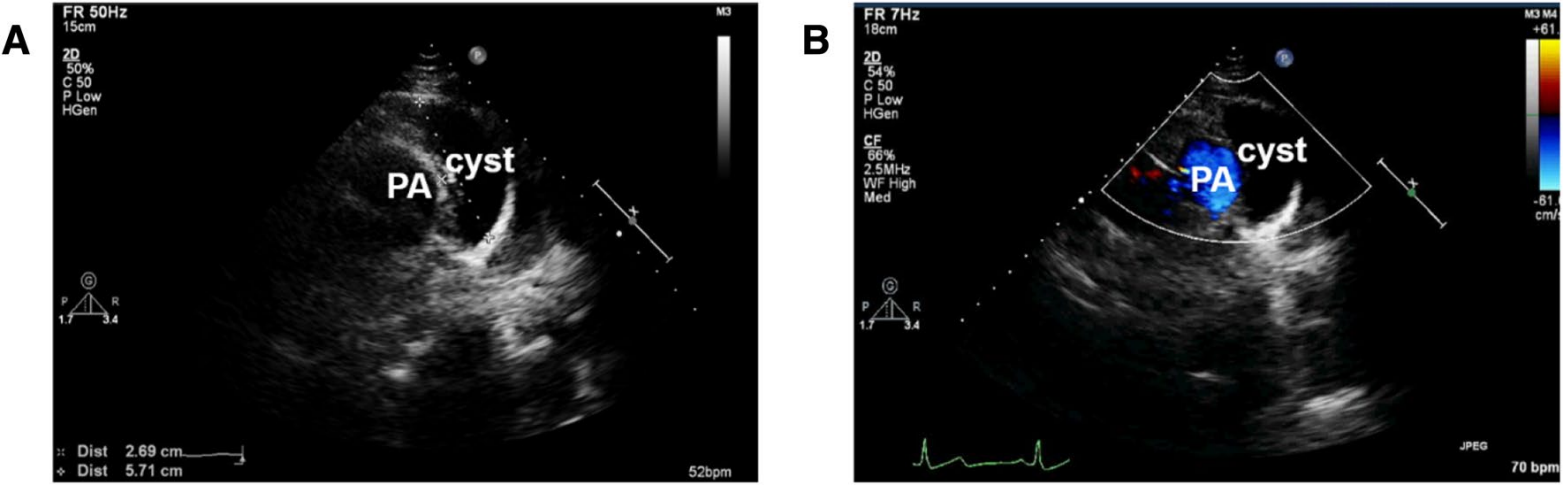
Echocardiographic images in a 45-year-old woman with a thymic cyst. **a** Nonstandard parasternal short-axis view of great arteries showed an echo-free structure adjacent to the pulmonary artery (PA); **b** CDFI demonstrated no blood flow within the cyst

**Fig. 3:**
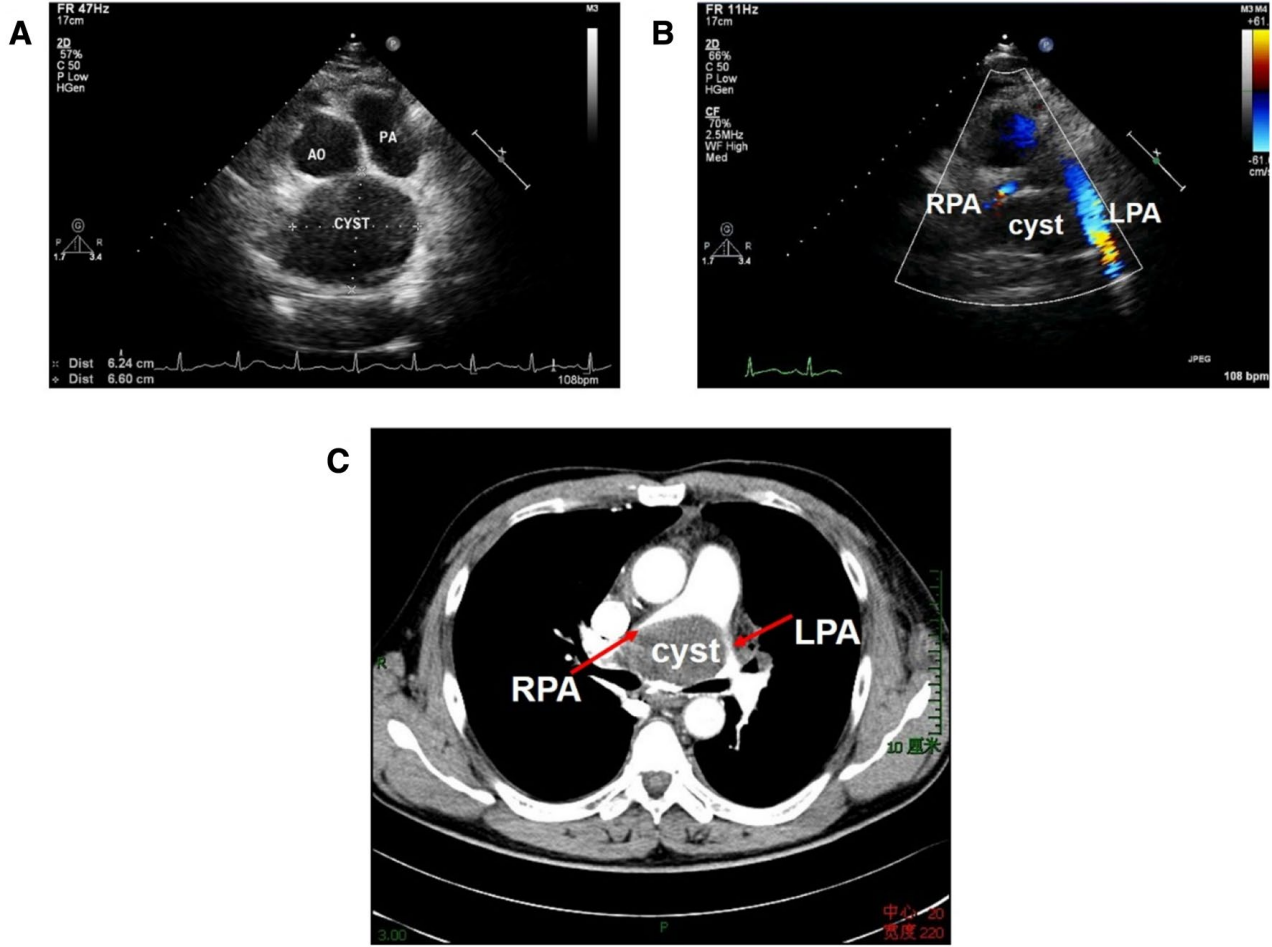
A 47-year-old man with a bronchogenic cyst, complaining of dyspnea and chest pain. **a** Nonstandard parasternal short-axis view of great arteries showed an echo-free structure in the upper region to the heart; **b** CDFI demonstrated accelerated blood flows in pulmonary branches resulting from the compression on bifurcation of the pulmonary artery; **c** Contrast-enhanced CT demonstrated the compression of the cyst. *PA* pulmonary artery, *LPA* left pulmonary artery, *RPA* right pulmonary artery

**Fig. 4:**
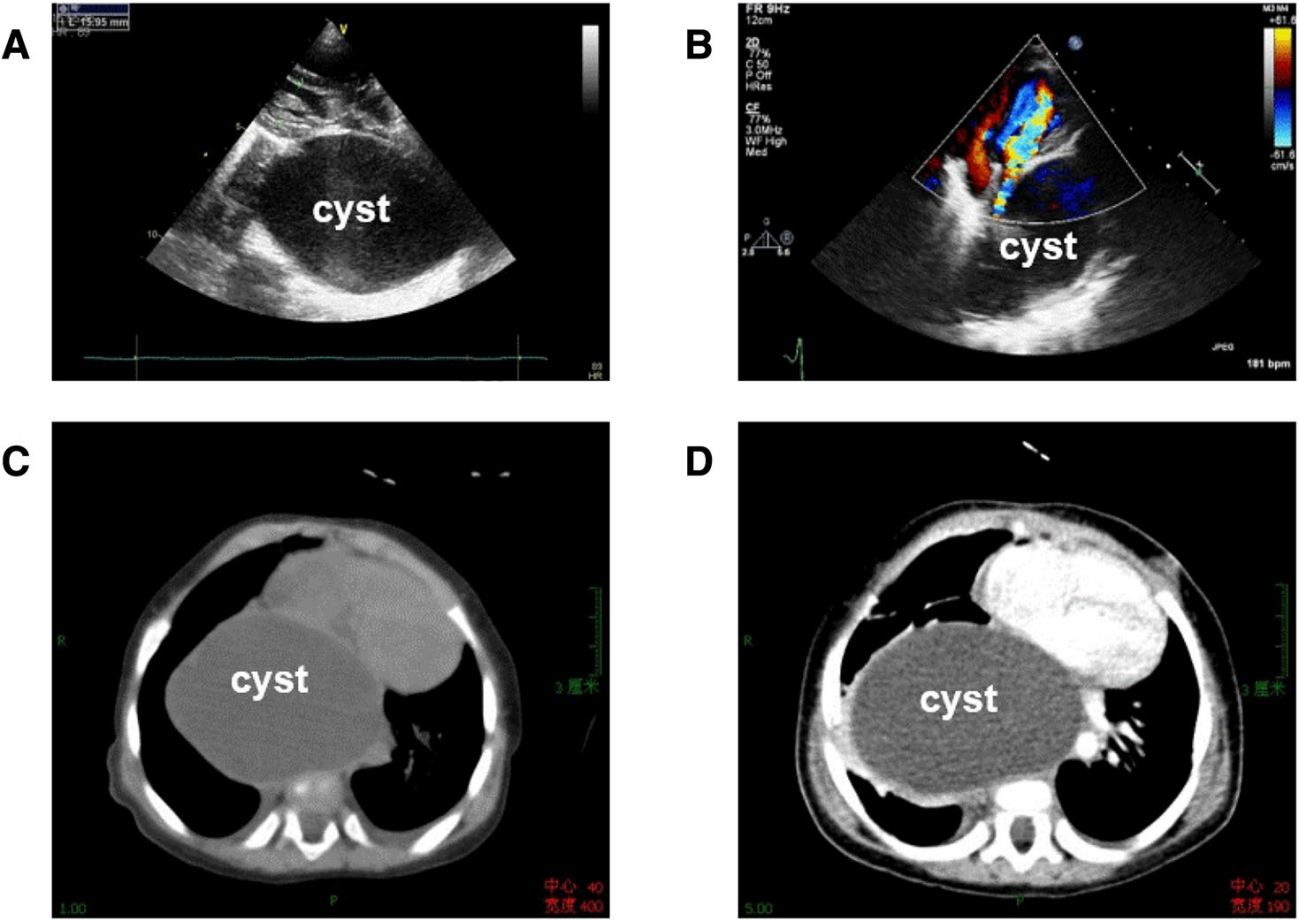
A 6-months-old female infant with a huge enteric cyst, repeatedly coughing for more than 10 days. **a** Parasternal long-axis view of the left ventricle showed a huge cystic structure behind the left atrium, compressing the atrium obviously; **b** the apex four-chamber view also indicated the compressive deformation of left atrium; **c**, **d** non-contrast CT (**c**) and contrast-enhanced CT (**d**) also demonstrated that a large cyst compressed the atrium and it had no blood perfusion

Among the patients diagnosed by echocardiography, all the cysts were presented as thin-walled, monolocular, echo-free structures adjacent to the heart in two-dimensional images and there was no obvious blood flow signal within the cysts in color doppler flow imaging (CDFI) (Fig. 2). In addition, 4 cysts showed compression on adjacent cardiac structures by echocardiography which were further confirmed by operation. The involved structures included right pulmonary artery, left atrium and bifurcation of pulmonary artery (Fig. 3), right atrium, and left atrium along with pulmonary vein (Fig. 4) in 1 case, respectively. Of all 43 cases, 4 patients had concomitant intracardiac abnormalities which were accurately diagnosed by echocardiography: 1 atrial septal defect, 1 anterior leaflet prolapse of mitral valve with moderate mitral regurgitation and 2 patent foramen ovale (Table 1).

## Discussion

A wide range of tissues exist in the regions around the heart; hence types of cystic lesions may occur in these areas. The most common pathological types of the PCLs are pericardial cysts, accounting for about half of all cases, and then followed by thymic cysts and bronchogenic cysts. A small part of cystic teratomas, enteric cysts and lymphangiomas can also be observed. Most PCLs occur in adults, except that the enteric cysts are usually diagnosed before the age of 1 year and may be associated with accompanying vertebral anomalies, including hemivertebrae, butterfly vertebra and anterior spina bifida [5]. The majority of the PCLs are usually asymptomatic or only with nonspecific symptoms, such as cough and chest pain [2, 3]. When the cysts are large and cause compression on adjacent structures, life-threatening symptoms may occur, such as syncope, pericardial tamponade or even sudden death [6–10].

PCLs are mostly detected by imaging examination and further confirmed by pathological results. Of the many imaging techniques, CT and MR are widely used in the diagnosis because their tomographic images can demonstrate the exact location of the lesions and the relationship to adjacent structures [11, 12]. On the contrary, echocardiography has specific limitations since the limited acoustic windows caused by the obstruction of the skeletons and pulmonary tissues [4, 12, 13]. However, with its widespread application in clinical practice, an increasing number of PCLs are detected by echocardiography [14]. As shown in this study, all patients undergoing CT or/and MR examination were exactly diagnosed. On the other hand, not all PCLs were diagnosed by echocardiography and about one third of the cases were missed the diagnosis.

In five predilection areas of these cysts, the missed diagnosis rates of echocardiography were not significantly different. It seemed that the detectable rate of echocardiography was irrelevant to the location of the cyst. We consider that the missed diagnosis may be due to the poor acoustic window, at the same time, it can’t be excluded that the missed diagnosis may be caused by the neglect of examiner since echocardiography examination is sonographer dependent. Therefore, we should go through all the sections carefully in echocardiography examination in order to avoid missed diagnosis caused by human factors [13].

All diagnosed cysts showed as thin-walled, monocular, echo-free structures and with no obvious blood flow signals in two-dimensional echocardiographic images. It is reported that three-dimensional echocardiography adds incremental value to two-dimensional echocardiography in the detection of PCLs, not only allowing accurate measurements of cyst size, but also better demonstrating the structure of the cyst [15, 16]. As mentioned above, the clinical features of the PCLs are nonspecific except the enteric cyst. Therefore, it is difficult to determine pathological types of the PCLs through clinical symptoms and echocardiographic features. At this time, the location of the cyst can be used for differential diagnosis [5, 17, 18]. Pericardial cysts are typically located at the right or left cardiophrenic angle and rarely in other areas [19]. Thymic cysts may occur at any part of the mediastinum and the most common location is in the anterior mediastinum [17]. Bronchogenic cysts commonly occur near the tracheal carina in the middle mediastinum [20]. The majority of cystic teratomas are in the anterior mediastinum and only a few locates in the posterior mediastinum [21]. Enteric cysts are usually located in the right posterior mediastinum [5]. In our study, in the left or right side of the heart, more than 50% of the cysts were pericardial cysts and a small number of them were thymic cysts and cystic teratoma. In the upper area adjacent to the heart, thymic cysts were the most common, followed by bronchogenic cysts and pericardial cysts. In addition, one case of pericardial cyst appeared in the anterior area of the heart. There were only 2 cysts located behind the heart, of which one was an enteric cyst (Fig. 3) and the other was a bronchogenic cyst.

Echocardiography is not only able to identify the cysts, but also can be used to assess their complications and the most important aspect of which is the compression on adjacent structures [6, 19, 22, 23]. Echocardiography can demonstrate corresponding distortion of cardiac chambers and great vessels [24]. In the present study, 4 cases showed compression on adjacent tissues, including left or right atrium, pulmonary artery and pulmonary veins. Maybe the relative low pressure of the atriums and pulmonary circulation contributed that they are easily to be compressed by large cysts. Furthermore, it has been reported that some other kinds of complications can be diagnosed by echocardiography occasionally, such as acute cardiac tamponade resulted from hemorrhagic pericardial cysts and evident cardiac displacement caused by a giant mediastinal cyst [7, 25].

In addition to the evaluation of cysts and related complications, echocardiography has an incomparable advantage in detecting intracardiac lesions together with the cysts than other imaging modalities. In this study, 4 patients with intracardiac lesions were identified by echocardiography. The combined lesions were not treated during the operation in 2 patients with PFO and one with MVP. The other female patient was admitted to hospital for thyroid disease and she was diagnosed with PCL and atrial septal defect (ASD) accidentally by echocardiography because of her chest pain. Then she underwent cyst resection and ASD closure simultaneously. The echocardiography findings were also confirmed by intraoperative and postoperative pathological results. We hypothesized that if the patient had not undergone echocardiography examination, the ASD would be missed the diagnosis since CT/MR were less valuable than echocardiography in detection of intracardiac lesions. If only the cyst was removed in the operation, the patient had to undergo another surgery for ASD. Therefore, it is necessary for patients diagnosed with PCLs by other imaging techniques to have a comprehensive echocardiographic examination for evaluating the structure and function of the heart before surgery.

Besides the important role in the diagnosis of the cysts, echocardiography can also be used in the treatment of cysts [26–28]. When a PCL is suspected, echocardiography-guided percutaneous aspiration may be an attractive option to confirm the diagnosis and treat the lesion [28]. Many studies have reported echocardiography-guided aspiration of large cysts and the compression symptoms were relieved after treatment [26–28].

## Limitations

Not all patients with PCLs have undergone echocardiography examination which are not included in our group, so there are limited numbers of cases in this study. Furthermore, some patients suspected with PCLs by echocardiography were asymptomatic and they followed conservative treatment without surgical resection. These patients were also excluded because the echocardiographic findings were not confirmed by pathological results.

## Conclusion

Multiple pathological types of cystic lesions may be encountered in echocardiography. The most common are pericardial cysts, followed by thymic cysts and bronchogenic cysts. The differential diagnosis of the types of PCLs can be made based on their locations. Echocardiography is a useful modality in the diagnosis of the PCLs, as well as in the evaluation of their complications and combined intracardiac lesions.
